# Correction: Extra-mitochondrial citrate synthase initiates calcium oscillation and suppresses age-dependent sperm dysfunction

**DOI:** 10.1038/s41374-019-0369-8

**Published:** 2020-01-06

**Authors:** Woojin Kang, Yuichirou Harada, Kenji Yamatoya, Natsuko Kawano, Seiya Kanai, Yoshitaka Miyamoto, Akihiro Nakamura, Mami Miyado, Yoshiki Hayashi, Yoko Kuroki, Hidekazu Saito, Yasuhiro Iwao, Akihiro Umezawa, Kenji Miyado

**Affiliations:** 1 Department of Reproductive Biology, National Research Institute for Child Health and Development, 2-10-1 Okura, Setagaya, Tokyo 157-8535 Japan; 2 Department of Perinatal Medicine and Maternal Care, National Center for Child Health and Development, 2-10-1 Okura, Setagaya, Tokyo 157-8535 Japan; 3 Institute for Environmental and Gender-Specific Medicine, Juntendo University Graduate School of Medicine, 2-1-1 Tomioka, Urayasu-City, Chiba 279-0021 Japan; 4 Department of Life Sciences, School of Agriculture, Meiji University, 1-1-1 Higashi-Mita, Tama-ku, Kawasakishi, Kanagawa 214-8571 Japan; 5 Department of Molecular Endocrinology, National Research Institute for Child Health and Development, 2-10-1 Okura, Setagaya, Tokyo 157-8535 Japan; 6 Life Science Center for Survival Dynamics, Tsukuba Advanced Research Alliance (TARA), University of Tsukuba, 1-1-1 Tennodai, Tsukuba, Ibaraki 305-8572 Japan; 7 Department of Genome Medicine, National Research Institute for Child Health and Development, 2-10-1 Okura, Setagaya, Tokyo 157-8535 Japan; 8 Division of Earth Science, Biology, and Chemistry, Graduate School of Sciences and Technology for Innovation, Yamaguchi University, 1677-1 Yoshida, Yamaguchi City, Yamaguchi 753-8511 Japan; 9 Present Address: Department of Molecular Pathology, Tokyo Medical University, 6-1-1 Shinjuku, Shinjuku, Tokyo 160-8402 Japan

**Keywords:** Ageing, Reproductive biology

**Correction to: Laboratory Investigation**

10.1038/s41374-019-0353-3

The article Extra-mitochondrial citrate synthase initiates calcium oscillation and suppresses age-dependent sperm dysfunction, written by Woojin Kang, Yuichirou Harada, Kenji Yamatoya, Natsuko Kawano, Seiya Kanai, Yoshitaka Miyamoto, Akihiro Nakamura, Mami Miyado, Yoshiki Hayashi, Yoko Kuroki, Hidekazu Saito, Yasuhiro Iwao, Akihiro Umezawa and Kenji Miyado, was originally published electronically on the publisher’s internet portal on 19th December 2019 without open access. With the authors’ decision to opt for Open Choice the copyright of the article changed on 9th January to © The Author(s) 2019 and the article is forthwith distributed under a Creative Commons Attribution 4.0 International License (https://creativecommons.org/licenses/by/4.0/), which permits use, sharing, adaptation, distribution and reproduction in any medium or format, as long as you give appropriate credit to the original author(s) and the source, provide a link to the Creative Commons licence, and indicate if changes were made.

The original version of this article was revised due to a retrospective Open Access order.

